# Epidemiological and clinical features of 2019 novel coronavirus diseases (COVID-19) in the South of Iran

**DOI:** 10.1186/s12879-020-05128-x

**Published:** 2020-06-18

**Authors:** Reza Shahriarirad, Zohre Khodamoradi, Amirhossein Erfani, Hamidreza Hosseinpour, Keivan Ranjbar, Yasaman Emami, Alireza Mirahmadizadeh, Mehrzad Lotfi, Babak Shirazi Yeganeh, Abolfazl Dorrani Nejad, Abdolrasool Hemmati, Mostafa Ebrahimi, Mohsen Moghadami

**Affiliations:** 1 Thoracic and Vascular Surgery Research Center, Shiraz University of Medical Sciences, Shiraz, Iran; 2 Student Research Committee, Shiraz University of Medical Sciences, Shiraz, Iran; 3 Department of Internal Medicine, Shiraz University of Medical Sciences, Shiraz, Iran; 4 Shiraz Geriatric Research Center, Shiraz University of Medical Sciences, Shiraz, Iran; 5 Department of Radiology, Medical Imaging Research Center, Shiraz University of Medical Sciences, Shiraz, Iran; 6 Non-communicable Diseases Research Center, Shiraz University of Medical Sciences, Shiraz, Iran; 7 Department of Pathology, School of Medicine, Shiraz University of Medical Sciences, Shiraz, Iran; 8 Vice Chancellor of Health Affairs, Shiraz University of Medical Sciences, Shiraz, Iran; 9 Department of Communicable Diseases, Shiraz University of Medical Science, Shiraz, Iran; 10 Clinical Microbiology Research Center, Shiraz University of Medical Sciences, Shiraz, 14336 – 71348 Iran

**Keywords:** COVID-19, Iran, Clinical characteristics

## Abstract

**Background:**

In March 2020, the WHO declared the novel coronavirus (COVID-19) outbreak a global pandemic. Although the number of infected cases is increasing, information about its clinical characteristics in the Middle East, especially in Iran, a country which is considered to be one of the most important focal points of the disease in the world, is lacking. To date, there is no available literature on the clinical data on COVID-19 patients in Iran.

**Methods:**

In this multicenter retrospective study, 113 hospitalized confirmed cases of COVID-19 admitted to university affiliated hospitals in Shiraz, Iran from February 20 to March 20 were entered in the study.

**Results:**

The mean age was 53.75 years and 71 (62.8%) were males. The most common symptoms at onset were fatigue (75: 66.4%), cough (73: 64.6%), and fever (67: 59.3%). Laboratory data revealed significant correlation between lymphocyte count (*P* value = 0.003), partial thromboplastin time (*P* value = 0.000), international normalized ratio (P value = 0.000) with the severity of the disease. The most common abnormality in chest CT scans was ground-glass opacity (77: 93.9%), followed by consolidation (48: 58.5%). Our results revealed an overall 8% (9 out of 113 cases) mortality rate among patients, in which the majority was among patients admitted to the ICU (5: 55.6%).

**Conclusion:**

Evaluating the clinical data of COVID-19 patients and finding the source of infection and studying the behavior of the disease is crucial for understanding the pandemic.

## Background

In late December 2019, China reported an outbreak of viral pneumonia in Wuhan, Hubei Province, China, which spread rapidly to other areas [1, 2]. It was revealed that the causative agent of the cluster of acute respiratory illness was an RNA enveloped beta coronavirus from the sarbecovirus subgenus of *Coronaviridae’s* family, which was termed the novel coronavirus disease 2019 (COVID-19) [3–6]. COVID- 19 counts as the third outbreak of betacoronaviruses in the twenty-first century, causing a public health crisis of global concern [7, 8]. Previous outbreaks of this viral family have been described in 2002 and 2012. The former was a respiratory disease identified as Severe Acute Respiratory Syndrome Coronavirus (SARS-CoV), involving 37 countries, and the latter, known as the Middle East Respiratory Syndrome Coronavirus (MERS-CoV) affected 27 countries. The overall mortality rate of these two epidemics of SARS-CoV and MERS-CoV was 10 and 37%, respectively [4, 9–12].

COVID-19 is a global concern and has become a significant health problem since the number of infected cases and affected countries has escalated rapidly [13]. On March 11, 2020, the World Health Organization (WHO) confirmed COVID-19 a pandemic. As of March 31, 2020, over 800,000 cases of COVID have been reported with a death toll of over 39,000 patients and only around 141,000 recovered cases in 199 countries and territories worldwide. Among the top-ranking countries, Iran was placed in seventh position with over 35,000 confirmed cases and over 2500 deaths, and only around 11,600 recovered cases [14, 15]. However, the actual number of cases may be much higher because of challenges in confirming the cases due to the limited PCR diagnostic test kits and available staff in the hospitals.

Based on the literature, the incubation period of the disease could be up to 14 days [16]. Most cases have mild symptoms of fever, cough, sore throat, and myalgia. However, some cases can present with severe conditions such as multiple organ failure, acute respiratory distress syndrome, pulmonary edema, and pneumonia [17–19]. Based on radiological findings in previous studies, the most frequent CT findings included bilateral pulmonary parenchymal ground-glass and consolidative pulmonary opacities, occasionally with a rounded morphology and a peripheral lung distribution [20, 21]. In respect to laboratory data, a decrease in the absolute value of lymphocytes in most patients can be found [22], indicating that the virus may mainly act on lymphocytes, especially T-cells. Damage to T lymphocytes can be a primary factor resulting in exacerbations of patients [23]. In clinical practice, a low absolute value of lymphocytes could assist as a reference index in diagnosing new cases of coronavirus infections. Due to the severity of the disease, with over 20% critical patients and mortality rate of about 3%, COVID-19 is a global health emergency [24]. Therefore, early detection and appropriate treatment of critical cases are of essential importance.

At present, there is a lack of information regarding the epidemiology and clinical features of COVID-19 patients in the Middle East, especially Iran, a country which is considered as one of the most important focal points of the disease throughout the world. Therefore, this study has been conducted to evaluate the clinical features of COVID-19 patients in Fars province, southern Iran.

## Methods

### Study design

The center for control and prevention of 2019 novel Coronavirus Disease (*COVID*-*19*) *was* established on February 20, 2020, to monitor the spread of the COVID*-*19 in Fars Province, Iran’s fourth most populated province. The center has been assigned to provide services to five hospitals affiliated by the Shiraz University of Medical Sciences. The approach to the disease was based on Iran national health guidelines, adapted from the WHO guidelines, and the latest studies on COVID*-*19 [25]. The incubation period of COVID-19 has been defined as the time of exposure to the onset of illness, which, based on reports from China and all over the world, was assumed to be between 3 to 14 days. A patient with symptoms of fever, rhinorrhea, cough, sore throat, and possibly respiratory distress is defined as suspected of having COVID-19, especially if there was a positive history of close contact with a highly suspected or confirmed COVID-19 patient, or having a travel history to a COVID-19 affected country or city [26]. Confirmed COVID-19 cases were admitted and quarantined in centers allocated for COVID-19 diagnosis and under the management of Shiraz University of Medical Sciences.

The severity of disease was based on the American Thoracic Society guidelines for community-acquired pneumonia as severe and non-severe, similar to other studies [27, 28], which in our research was the reference for intensive care unit (ICU) or non-ICU admission.

### Data collection

The epidemiological and clinical (including clinical records, laboratory data, as well as data regarding the chest high resolution CT (HRCT) scans) data of all confirmed COVID-19 patients from February 20, 2020, to March 20, 2020, in allocated centers for COVID-19 diagnosis and under the management of Shiraz University of Medical Sciences were collected. Data were recruited consecutively from the available patients and also extracted from the hospital records in the cases of discharged patients. All physical examinations were performed by a solo physician. Furthermore, the primary chest CT scan was reviewed independently by an unaccompanied trained specialized radiologist who was blind to the primary impression, clinical symptoms and the patient’s outcome.

### Ethical statement

The ethics committee of Shiraz University of Medical Sciences approved the current study with the ethical code number: IR.SUMS.REC.1398.1378. Confidentiality of patient information was guaranteed and protected by recording only birth date, gender, marital status, occupation, comorbid disease. The goal of the study was explained to the patients and written informed consents were obtained from them.

### Laboratory confirmation of COVID-19

Real-time reverse transcriptase-polymerase chain reaction (RT-PCR) was used to confirm suspected cases. RT-PCR assays were performed following the protocol established by the WHO [25]. Nasopharyngeal and oropharyngeal swab samples were collected and tested for SARS-CoV-2 for each patient. Under a biosafety cabinet and according to laboratory biosafety guidelines, the RNAs were extracted, using QIAamp™ viral RNA mini kit from Qiagen™ according to the manufacturer’s instructions. With E-gene and Rdrp-gene probe/primer and superscript™ III platinum, one-step qRT-PCR kit of Invitrogen company mixtures was prepared. The mixtures transferred to Roche Light cycler™ 96 and Applied Biosystem ABI step one plus™ real time thermal cyclers with positive control and no template control (NTC) as well as an internal control. After 45 cycles the produced graphs were observed, any rise after the noise and before cycle 32 was considered as positive for SARS-COV 2 [29, 30].

### Statistical analysis

The collected data was summarized as means (±SD: standard deviation) or medians (with interquartile ranges). For particular variables, the percentage of patients in each group was calculated. Unpaired Student’s t-test, chi-square test, or Fisher’s exact test was used to compare the clinical characteristics of COVID-19 patients as appropriate. A P. value of less than 0.05 was considered as indicating statistical significance. All the statistical analyses were performed by the Statistical Package for Social Sciences (SPSS Inc., Chicago, Illinois, USA) version 26.0.

## Results

### Presenting characteristics

The study population consisted of 113 confirmed COVID-19 cases, with a mean age of 53.75 years (SD = 16.58; range 20–99). The patients consisted of 71 (62.8%) males and 42 (37.2%) females. Among the patients in our study, 11 (9.7%) were admitted to intensive care units due to the severity of their disease. The average time between the initiation of symptoms until hospital admission was 5.63 days, and the average duration of hospitalization was 6.20 days. The average number of days from the start of symptoms until the development of dyspnea, and progression to acute respiratory distress syndrome (ARDS) was 5.63, 0.5, and 2 days, respectively. Among the patients in our study 44 (38.9%) had one or more coexisting medical conditions alongside COVID-19; the most frequent were hypertension (22: 19.5%), diabetes (16: 14.2%), and cardiovascular diseases (16: 14.2%) (Table 1).

**Table 1:** Clinical and demographic features of COVID-19 patients in Shiraz, South of Iran

**Variable**	**Total** (%) *n = 113*	**Severe** *n = 11*	**Non-severe** *n = 102*	***P*.value**	**Death***n = 9*	**Live***n = 104*	***P*.value**
**Age (years)**							
20–34	13 (11.5)	1 (7.7)	12 (92.3)	0.750	1 (7.7)	12 (92.3)	0.899
35–49	36 (31.9)	2 (5.6)	34 (94.4)		2 (5.6)	34 (94.4)	
50–64	36 (31.9)	5 (13.9)	31 (86.1)		3 (8.3)	33 (91.7)	
65–74	14 (12.4)	2 (14.3)	12 (85.7)		1 (7.1)	13 (92.9)	
≥ 75	14 (12.4)	1 (7.1)	13 (92.9)		2 (14.3)	12 (85.7)	
**Sex**							
Male	71 (62.8)	7 (9.9)	64 (90.1)	0.750	5 (7)	66 (93)	0.725
Female	42 (37.2)	4 (9.5)	38 (90.5)		4 (9.5)	38 (90.5)	
**Occupation**							
Healthcare worker	3 (2.8)	0 (0)	3 (100)	1.000	0 (0)	3 (100)	1.000
Non-healthcare worker	103 (97.2)	9 (8.7)	94 (91.3)		9 (8.7)	94 (91.3)	
**History of Contact with infected cases**	19 (16.8)	0 (0)	19 (100)	0.206	0 (0)	19 (100)	0.353
**History of travelling**	30 (26.5)	3 (10)	27 (90)	1.000	2 (6.7)	28 (93.3)	1.000
**Comorbid Disease**							
Hypertension	22 (19.5)	5 (22.7)	17 (77.3)	0.037	2 (9.1)	20 (90.9)	1.000
Diabetes	16 (14.2)	3 (18.8)	13 (81.3)	0.188	2 (12.5)	14 (87.5)	0.613
Cardiovascular disease	16 (14.2)	4 (25)	12 (75)	0.049	2 (12.5)	14 (87.5)	0.613
Malignancy	1 (0.9)	0 (0)	1 (100)	1.000	0 (0)	1 (100)	1.000
Asthma	7 (6.2)	1 (14.3)	6 (85.7)	0.522	1 (14.3)	6 (85.7)	0.450
Chronic obstructive Pulmonary disease	9 (8.0)	1 (11.1)	8 (88.9)	1.000	1 (11.1)	8 (88.9)	0.540
Chronic kidney disease	6 (5.3)	0 (0)	6 (100)	1.000	0 (0)	6 (100)	1.000
Other Immunosuppressive diseases	2 (1.8)	0 (0)	2 (100)	1.000	0 (0)	2 (100)	1.000
**Symptoms at onset of illness**							
Fever	67 (59.3)	5 (7.5)	62 (92.5)	0.350	3 (4.5)	64 (95.5)	0.156
Cough	73 (64.6)	5 (6.8)	68 (93.2)	0.193	5 (6.8)	68 (93.2)	0.718
Fatigue	75 (66.4)	10 (13.3)	65 (86.7)	0.096	8 (10.7)	67 (89.3)	0.268
Sputum production	24 (21.4)	2 (8.3)	22 (91.7)	1.000	4 (16.7)	20 (83.3)	0.097
Dyspnea	58 (51.3)	8 (13.8)	50 (86.2)	0.205	4 (6.9)	54 (93.1)	0.738
Chest pain	43 (38.1)	6 (14)	37 (86)	0.328	3 (7)	40 (93)	1.000
Chills	67 (59.3)	4 (6)	63 (94)	0.114	3 (4.5)	64 (95.5)	0.153
Hemoptysis	7 (6.2)	0 (0)	7 (100)	1.000	0 (0)	7 (100)	1.000
Rhinorrhea	26 (23.0)	1 (3.8)	25 (96.2)	0.452	2 (7.7)	24 (92.3)	1.000
Sore throat	36 (31.9)	3 (8.3)	33 (91.7)	1.000	2 (5.6)	34 (94.4)	0.716
Abdominal pain	24 (21.2)	2 (8.3)	22 (91.7)	1.000	2 (8.3)	22 (91.7)	1.000
Diarrhea	25 (22.1)	1 (4)	24 (96)	0.451	2 (8)	23 (92)	1.000
Nausea	48 (42.5)	5 (10.4)	43 (89.6)	1.000	5 (10.4)	43 (89.6)	0.491
Vomiting	29 (25.7)	2 (6.9)	27 (93.1)	0.725	4 (13.8)	25 (86.2)	0.234
Anorexia	75 (66.4)	9 (12)	66 (88)	0.329	8 (10.7)	67 (89.3)	0.268
Myalgia /Arthralgia	69 (61.1)	7 (10.1)	62 (89.9)	1.000	5 (7.2)	64 (92.8)	0.734
Headache	60 (53.1)	5 (8.3)	55 (91.7)	0.753	6 (10)	54 (90)	0.498
Dizziness/ Vertigo	45 (39.8)	7 (15.6)	38 (84.4)	0.111	6 (13.3)	39 (86.7)	0.152
Conjunctival congestion	17 (15.0)	2 (11.8)	15 (88.2)	0.670	2 (11.8)	15 (88.2)	0.622
**Physical exam on admission**							
**Temperature**							
< 37.3	77 (68.1)	8 (10.4)	69 (89.6)	0.005	6 (7.8)	71 (92.2)	0.002
37.3–38	24 (21.2)	0 (0)	24 (100)		0 (0)	24 (100)	
38.1–39	11 (9.7)	2 (18.2)	9 (81.8)		2 (18.2)	9 (81.8)	
> 39	1 (0.9)	1 (0.9)	0 (0)		1 (100)	0 (0)	
**Heart Rate**							
> 100 (beats/min)	12 (10.6)	1 (7.7)	12 (92.3)	1.000	1 (7.7)	12 (92.3)	1.000
**Respiratory Rate**							
> 24(breaths/min)	2 (1.8)	1 (50)	1 (50)	0.186	1 (50)	1 (50)	0.154
**Blood Pressure**							
Normal	36 (32.4)	4 (11.1)	32 (88.9)	0.738	4 (11.1)	32 (88.9)	0.617
Elevated	7 (6.3)	7 (10)	63 (90)		5 (7.1)	65 (92.9)	
**Saturation of O**_**2**_							
< 90	39 (34.5)	7 (17.9)	32 (82.1)	0.046^a^	4 (5.4)	70 (94.6)	0.271
≥ 90	74 (65.5)	4 (5.4)	70 (94.6)		5 (12.8)	34 (87.2)	
**Lung Auscultation**							
Respiratory Rales	27 (23.9)	7 (25.9)	20 (74.1)	0.004^a^	3 (11.1)	24.(88.9)	0.444
Respiratory Wheeze	8 (7.1)	0 (0)	8 (100)	1.000	0 (0)	8 (100)	1.000
Respiratory Stridor	10 (8.8)	1 (10)	9 (90)	1.000	1 (10)	9 (90)	0.580
**Other Infection Signs**							
Throat Congestion	17 (15.0)	4 (23.5)	13 (76.5)	0.060	1 (5.9)	16 (94.1)	1.000
Swelling of tonsils	8 (7.1)	1 (12.5)	7 (87.5)	0.571	0 (0)	8 (100)	1.000
Lymphadenopathy	3 (2.7)	1 (33.3)	2 (66.7)	0.267	0 (0)	3 (100)	1.000
Rash	6 (5.3)	0 (0)	6 (100)	1.000	1 (16.7)	5 (83.3)	0.377
**Treatment**							
Anti-Viral therapy	113 (100.0)	11 (9.7)	102 (90.3)	NA	9 (8)	104 (92)	NA
Antibiotic therapy	112 (99.1)	11 (9.8)	101 (90.2)	1.000	9 (8)	103 (92)	1.000
Use of Corticosteroid	5 (4.4)	2 (40)	3 (60)	0.074	1 (20)	4 (80)	0.345
**Oxygen support**							
Nasal cannula	31 (27.4)	1 (3.2)	30 (96.8)	0.285	1 (3.2)	30 (96.8)	0.440
Noninvasive ventilation or high flow mask	11 (9.8)	6 (54.5)	5 (45.5)	0.000^a^	1 (9.1)	10 (90.9)	1.000
Invasive mechanical ventilation	2 (1.8)	2 (100)	0.000	0.009^a^	2 (100)	0 (0)	0.006^a^
**Prognosis**							
Hospitalization	29 (25.7)	2 (6.9)	27 (93.1)	0.000^a^	0 (0)	29 (100)	0.000^a^
Discharge with continued OPD treatment	7 (6.2)	1 (14.3)	6 (85.7)		0 (0)	7 (100)	
Total Recovery	68 (60.2)	3 (4.4)	65 (95.6)		0 (0)	68 (100)	
Death in course of Hospitalization	9 (8)	5 (55.6)	4 (44.4)		9 (100)	0 (0)	

^a^ indicator of significant correlation; NA: not applicable

Among the hospitalized patients, the most common symptoms at onset of disease were fatigue (66:4%), cough (64.6%), and fever (59.3%); while the less common were hemoptysis (6.2%), and conjunctival congestion (15%) (Table 1).

### Vital signs and physical examination

Based on the patients’ vital signs on admission, 77 (68.1%) of our patients had no fever. Also, 12 (10.6%) had elevated heart rates (> 100/min), 2 (1.8%) had elevated respiratory rate (> 24/min), and 7 (6.3%) had elevated blood pressure on admission. These values did not differ between severe and non-severe patients and were all lower among the deceased patients, although not significant. The oxygen saturation of the patients was also measured on admission, which showed 39 (34.5%) of the patients had an O_2_ saturation of less than 90%. Also, the mean saturation of O_2_ was significantly lower among the ICU admitted patients (*P* = 0.001) and was also significantly lower among the deceased patients (*P* < 0.05) (Table 2).

**Table 2:** Vital signs at admission of COVID-19 patients in Shiraz, South of Iran

**Vital signs on admission**	**Mean Total (±SD)***n = 113*	**Mean (±SD)**		***P*.value**	**Mean (±SD)**		***P*.value**
		**Severe***n = 102*	**Non-Severe***n = 11*		**Death***n = 9*	**Live***n = 104*	
Systole Blood Pressure (mmHg)	129.74 (±95.50)	116.91 (±13.94)	131 (±100.42)	0.641	117.44 (±15.05)	130.82 (±99.46)	0.689
Diastole Blood Pressure (mmHg)	74.56 (±10.68)	77.00 (±13.12)	74.29 (±10.42)	0.427	74 (±12.28)	74.61 (±10.58)	0.871
O2 Saturation	90.12 (±5.66)	84.64 (±8.91)	90.71 (±4.90)	0.001^a^	86.00 (±7.58)	90.47 (±5.363)	0.022^a^
Respiratory rate	18.99 (±12.88)	19.18 (±2.994)	18.97 (±7.02)	0.922	18.89 (±2.759)	19 (±6.979)	0.962
Heart rate	85.21 (±12.88)	84.60 (±13.53)	85.27 (±12.89)	0.875	79.88 (±16.47)	85.63 (±12.57)	0.226
Temperature	36.90 (±3.56)	37.34 (±1.06)	36.86 (±3.73)	0.673	37.47 (±1.138)	36.85 (±3.698)	0.623

^a^ indicator of significant correlation

In terms of the lung examinations, only seven patients (25.9%) had significant rales, which were significantly correlated with the severity of the disease. Among the other signs of infection, the most common were throat congestion (17: 15%), and swelling of the tonsils (8:7.1%) (Table 1).

### Laboratory findings

Numerous variations in laboratory findings were seen among the severe and non-severe, as well as the deceased and living patients (Table 3). ICU admitted patients who had significantly higher lymphocyte count than non-ICU patients, although this correlation was not significant with the mortality rate of the patients in our study. The severe group and the deceased group had higher levels of white blood cells and neutrophil count. Although lower levels of hemoglobin, hematocrit, and platelet count were detected compared to the non-severe and living groups, these differences were not statistically significant. Based on coagulation tests, there were significantly higher international normalized ratio (INR) levels and prolonged partial thromboplastin time (PTT) among the severe and non-severe group (*P* < 0.001); however, the significance of this finding among the deceased group was only valid for INR (P < 0.001).

**Table 3:** Laboratory features of COVID-19 patients in Shiraz, South of Iran; presented as either mean ± SD, or frequency

**Laboratory Findings**	**Normal Value**	**All patients** *n = 113*	**Sever** *n = 11*	**Not Sever** *n = 102*	***P*.value**	**Dead** *n = 9*	**Live** *n = 104*	***P*.value**
**White blood cell count**	3.5–9.5	6.06 (±2.50)	6.62 (±2.68)	6.00 (±2.49)	0.438	6.83 (±2.94)	6.00 (±2.47)	0.341
Leukopenia		10 (9)	0 (0)	10 (100)	0.424	1 (10)	9 (90)	0.484
Normal		89 (80.2)	9 (10.1)	80 (89.9)		6 (6.7)	83 (93.3)	
Leukocytosis		12 (10.8)	2 (16.7)	10 (83.3)		2 (16.7)	10 (83.3)	
**Neutrophil count (**^**a**^**10**^**9**^**/L)**	1.8–6.3	4.60 (±2.2)	10.33 (±0)	4.37 (± 1.93)	0.006	6.49 (± 3.47)	4.26 (± 1.83)	0.064
1.8–6.3		20 (76.9)	0 (0)	20 (100)	0.231	2 (10)	18 (90)	0.218
> 6.3		6 (23.1)	1 (16.7)	5 (83.3)		2 (33.3)	4 (66.7)	
**Lymphocyte count (**^**a**^**10**^**9**^**/L)**	1.1–3.2	1.16 (±0.66)	1.37 (± 0.62)	1.14 (± 0.66)	0.386	1.09 (±0.64)	1.16 (±0.66)	0.791
< 1500		14 (12.6)	4 (28.6)	10 (71.4)	0.030^a^	3 (21.4)	11 (78.6)	0.141
1.5–3.2		71 (64)	4 (5.6)	53 (94.4)		4 (5.6)	67 (94.4)	
> 3.2		26 (23.4)	3 (11.5)	23 (88.5)		2 (7.7)	24 (92.3)	
**Monocyte count (**^**a**^**10**^**9**^**/L)**	0.1–0.6	0.49 (±0.34)	0.46 (± 0)	0.50 (±0.38)	0.939	0.46 (± 0)	0.50 (± 0.38)	0.939
Normal		5 (83.3)	1 (20)	4 (80)	1.000	1 (20)	4 (80)	1.000
Increase Monocyte count		1 (16.6)	0 (0)	1 (100)		0 (0)	1 (100)	
**Hemoglobin (g/L)**	12–17.5	13.54 (±2.39)	13.28 (±1.53)	13.57 (±2.47)	0.706	13.02 (±2.26)	13.58 (±2.41)	0.500
**Hematocrit (%)**	36–54	40.42 (±4.69)	37.05 (±10.81)	40.66 (±4.29)	0.301	40.02 (±6.6)	40.50 (± 4.3)	0.838
**Platelet count**	150–450	220.92 (±105.66)	220 (±108.82)	220.98 (±105.87)	0.985	199.55 (±104.24)	222.85 (±106.09)	0.791
Thrombocytopenia		17 (15.6)	2 (11.8)	15 (88.2)	0.676	1 (5.9)	16 (94.1)	0.927
Normal		80 (73.4)	7 (8.8)	73 (91.3)		7 (8.8)	73 (91.3)	
Thrombocytosis		12 (11.0)	2 (16.7)	10 (83.3)		1 (8.3)	11 (91.7)	
**Prothrombin Time (S)**	9.4–13.5	15.35 (±2.38)	16.30 (±2.39)	15.25 (±2.37)	0.167	16.70 (± 1.54)	15.23 (± 2.41)	0.077
Normal		25 (22.1)	2 (8)	23 (92)	0.796	0 (0)	25 (100)	0.249
Prolonged		88 (77.9)	9 (10.2)	79 (89.8)		9 (10.2)	79 (89.8)	
**Partial Thromboplastin Time (S)**	25–36.5	37.61 (±8.49)	37.81 (±12.36)	37.59 (±8.02)	0.934	35.11 (± 10.48)	37.84 (± 8.31)	0.357
Decreased		1 (0.9)	2 (100)	0 (0)	0.000^a^	1 (50)	1 (50)	0.100
Normal		57 (53.3)	2 (3.6)	54 (96.4)		4 (7.1)	52 (92.9)	
Prolonged		49 (45.8)	7 (14.3)	42 (85.7)		4 (8.2)	45 (91.8)	
**International Normalized Ratio**	0–1.1	1.32 (±0.23)	1.85 (±0.49)	1.28 (±0.15)	0.000^a^	1.64 (± 0.32)	1.25 (± 0.13)	0.000^a^
**Blood urea nitrogen (mg/dL)**	7–20	16.80 (±5.81)	17.34 (±6.57)	16.75 (±5.76)	0.762	17.67 (± 16.73)	16.73 (± 5.68)	0.663
≤ 20		82 (76.6)	8 (9.8)	74 (90.2)	1.000	6 (7.3)	76 (92.7)	1.000
> 20		25 (23.4)	2 (8)	23 (92)		2 (8)	23 (92)	
**Creatinine (mg/dL)**	0.6–1.2	1.13 (±0.35)	1.22 (±0.38)	1.12 (±0.35)	0.418	1.02 (±0.29)	1.14 (±0.36)	0.386
≤ 1.2		69 (64.5)	6 (8.7)	63 (91.3)	0.741	6 (8.7)	63 (91.3)	0.709
> 1.2		38 (35.5)	4 (10.5)	34 (89.5)		2 (5.3)	36 (94.7)	
**Sodium (mmol/L)**	135–145	137.7(±5.12)	137.13 (±4.53)	137.73 (±5.21)	0.717	138.91 (±5.74)	137.56 (±5.09)	0.478
**Potassium (mmol/L)**	3.5–5	4.1(±0.6)	3.84 (±0.46)	4.08 (±0.57)	0.195	4.34 (± 0.68)	4.04 (0.56)	0.478
**Lactate dehydrogenase (U/L)**	0–250	686.23 (±509.39)	–	686.23 (± 509.39)	NA	1116.5 (± 1059.95)	628.86 (± 431.66)	0.214
**Aspartate aminotransferase (U/L)**	15–40	47.00 (±30.86)	49.00 (± 0)	47.00 (± 33.33)	1.000	47 (± 0)	47 (± 33.33)	1.000
**Alanine aminotransferase (U/L)**	9–50	35.87 (±36.48)	32.00 (± 0)	36.42 (± 39.36)	0.920	32.00 (± 0)	36.42 (±39.36)	0.920
**Albumin (g/L)**	3.4–5.4	3.90 (±0.46)	3.90 (±0)	3.90 (±0.47)	0.986	3.90 (±0.20)	3.90 (± 0.49)	0.974
**Total bilirubin (mmol/L)**	0–1.4	0.69 (±0.30)	0.64 (±0)	0.69 (±0.31)	0.866	0.38 (±0.21)	0.73 (± 0.29)	0.063
**Direct Bilirubin (mmol/L)**	0–0.3	0.34 (±0.16)	0.31(± 0)	0.34 (±0.16)	0.846	0.23 (± 0.10)	0.35 (±0.16)	0.333
**C reactive Protein (mg/L)**	0–8	34.32 (±20.40)	44.00 (±24.74)	33.33 (±19.79)	0.116	35.00 (± 25.27)	34.28 (±20.23)	0.934
< 8		9 (8.3)	0 (0)	9 (100)	0.127	0 (0)	9 (100)	0.616
8–50		72 (66.7)	5 (6.9)	67 (93.1)		5 (6.9)	67 (93.1)	
≥ 50		27 (25)	5 (18.5)	22 (81.5)		1 (3.7)	26 (96.3)	
**Erythrocyte sedimentation rate (mm/h)**	0–20	45.05 (±21.93)	45.66 (±22.39)	34 (±0)	0.619	41.50 (± 24.89)	46.00 (± 21.93)	0.726
< 20		1 (0.9)	0 (0)	1 (100)	1.000	0 (0)	1 (100)	1.000
≥ 20		18 (15.9)	1 (5.6)	17 (94.4)		4 (22.2)	14 (77.8)	
**PH**	7.35–7.45	7.35 (0.29)	7.41 (±0.05)	7.33 (±0.32)	0.567			
Acidosis		8 (7.1)	1 (16.7)	5 (83.3)	0.727	0 (0)	6 (100)	0.394
Normal		28 (24.8)	5 (17.9)	23 (82.1)		5 (17.9)	23 (82.1)	
Alkaline		3 (2.7)	0 (0)	3 (100)		1 (33.3)	2 (66.7)	
**Arterial Blood Gas**								
PCO2	35–48	44.08 (±10.73)	42.45 (± 6.61)	44.26 (± 11.11)	0.615	46.20 (± 15.27)	43.89 (± 10.33)	0.563
PO2	40	31.24(±13.74)	38.88 (±17.50)	30.35 (±13.06)	0.051	47.40 (± 27.23)	29.73 (10.83)	0.000^a^
HCO3	35–48	25.62 (±3.58)	25.91 (±3.25)	25.58 (±3.63)	0.789	25.82 (± 4.35)	25.60 (± 3.53)	0.868

NA: Not applicable; ^a^ indicator of significant correlation

Among the biochemical tests, the severe group showed higher blood urea nitrogen (BUN), creatinine, aspartate aminotransferase (AST), C reactive protein, and erythrocyte sedimentation rate (ESR) compared to the non-severe group, although it was not significant (Table 3).

The neutrophil to lymphocyte ratio (NLR) was calculated and compared based on the severity and mortality in the patients in our study. Results showed a significantly higher NLR among the ICU admitted group (severe group) and the expired group (*P* = 0.007 and 0.01, respectively). This difference was also significant among patients above 50 years of age (*P* = 0.023 and 0.021). However, the average NLR among patients above 50 years with ICU admission was 11.46 compared to 4.92 for above 50 years without ICU admission. For patients below 50 years of age, an average of 5.82 vs. 3.46 was calculated for the deceased and living, respectively (*P* > 0.05).

### Radiological findings

**Fig. 1:**
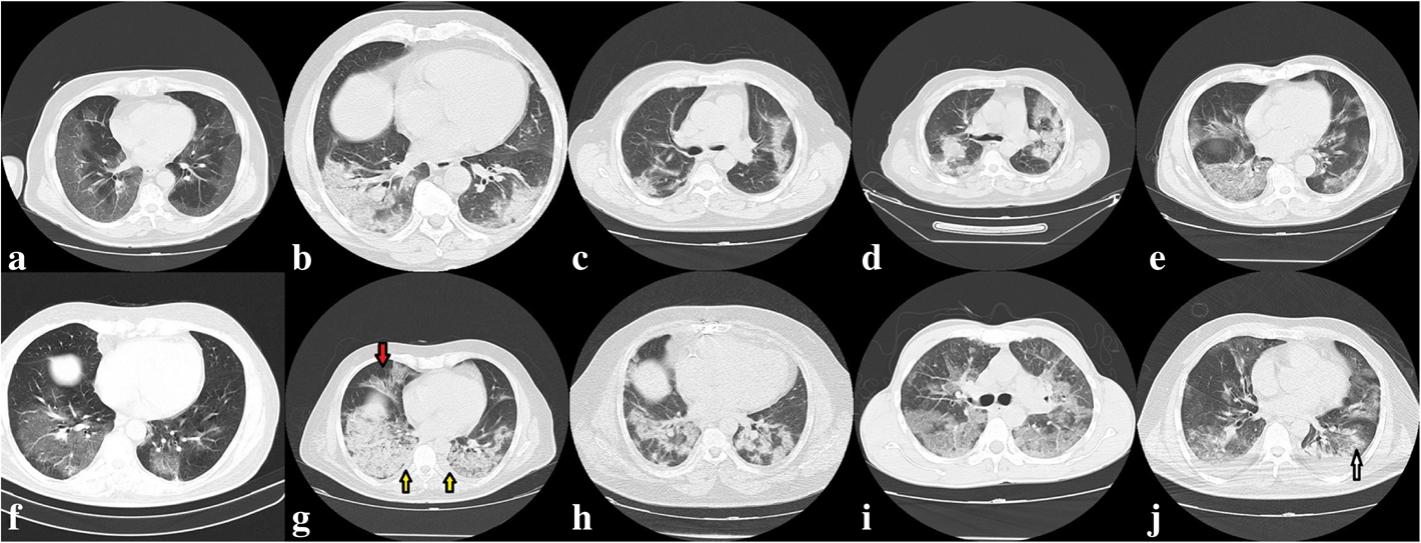
(**a**) Axial CT scans from an above 60 year-old male; Selected cut from non-contrast chest CT of lung window. Sub pleural crescent-shaped Ground-glass opacities as well as smooth interlobular septal thickening can be seen in both lungs, involving mostly peripheral zone; (**b**) Axial CT scans from an above 60 year-old male; selected image from non-contrast chest CT scans, lung window. Extensive consolidation with can be seen in both lower lobes with air bronchograms; (**c**) and (**d**) Axial CT scans from an above 50 year-old male at the level of carina; (**C**) Day 5 after symptom onset: patchy consolidation affecting the bilateral, peripheral lung parenchyma and (**d**) Day 7: expansion of consolidation in both lungs, as well as ground glass opacities in right side; (**e**) Axial CT scans from an above 60 year-old male; selected image from non-contrast chest CT scans, lung window. Mixed consolidation and ground glass opacities can be seen in both lower lobes, right middle lobe and lingula of left upper lobe; (**f**) and (**g**) Axial CT scans from an above 50 year-old male; selected image from non-contrast chest CT scans, lung window, (**f**) Day 3 after symptom onset: ground glass opacities in both lower lobe associated with mal focus of consolidation and (**g**) Day 7: expansion of consolidation in both lungs, as well as GGO in right middle lobe (black arrow); Mild pleural effusion is seen bilaterally (yellow arrows); (**h**) Axial CT scans from an above 50-year old female; selected image from non-contrast chest CT scans, lung window. Multiple patchy consolidation in both lower lobe; (**i**) Axial CT scans from an under 50 year-old male; selected image from non-contrast chest CT scans, lung window. Crazy-paving pattern (GGO with superimposed inter- and intralobular septal thickening) are seen bilaterally; (**j**) Axial CT scans from an above 50 year-old male; selected image from non-contrast chest CT scans, lung window. Ground-glass opacities affecting the bilateral lung field, reverse halo sign (ground-glass opacity surrounded by denser consolidation of crescentic shape) in left lower lobe (arrow), Pleural effusion is seen bilaterally

Radiological evaluation revealed 4 (4.9%) patients with normal CT scans, none of whom were among the severe or mortality group. These patients were dominantly male (75%) and under 50 years of age, although no significant correlation was achieved between gender and age with normal CT finding. The most common abnormality was ground-glass opacity (77: 93.9%), followed by consolidation (48: 58.5%). Also, radiological findings of crazy paving were significantly more frequent in non-severe and living patients (*P* < 0.05) (Table 4 and Fig. 1).

**Table 4:** Radiological findings of CT-scan of COVID-19 Patients in Shiraz, Fars

**Variable**	**Total (%)***n = 113*	**Severe** *n = 11*	**Non-severe** *n = 102*	***P*.value**	**Death***n = 9*	**Live***n = 104*	***P*.value**
**Involvement**							
Normal	4 (4.9)	0 (0)	4 (100)	0.519	0 (0)	4 (100)	0.728
Unilateral	8 (9.8)	0 (0)	8 (100)		1 (12.5)	7 (87.5)	
Bilateral	70 (85.4)	7 (10)	63 (90)		5 (7.1)	65 (92.9)	
**Distribution**							
Diffuse	9 (11)	3 (33.3)	6 (66.7)	0.019*	4 (44.4)	5 (55.6)	0.000*
Random (Peripheral and peribronchial)	18 (22)	3 (16.7)	15 (83.3)		1 (5.6)	17 (94.4)	
Peripheral	50 (61)	1 (2)	49 (98)		1 (2)	49 (98)	
Peribronchial	1 (1.2)	0 (0)	1 (100)		0 (0)	1 (100)	
**Ground Glass Opacity**	77 (93.9)	7 (9.1)	70 (90.9)	1.000	6 (7.8)	71 (92.2)	1.000
**Crazy Paving**	40 (48.8)	7 (17.5)	33 (82.5)	0.005*	6 (15)	34 (85)	0.011*
**Reverse halo**	10 (12.2)	0 (0)	10 (100)	0.589	1 (10)	9 (90)	0.554
**Reticular opacity**	8 (9.8)	1 (12.5)	7 (87.5)	0.527	0 (0)	8 (100)	1.000
**Consolidation**	48 (58.5)	4 (8.3)	44 (91.7)	1.000	3 (6.3)	45 (93.8)	0.688
**Centrilobular nodule**	2 (2.4)	0 (0)	2 (100)	1.000	0 (0)	2 (100)	1.000
**Solid Nodule**	9 (11)	0 (0)	9 (100)	1.000	0 (0)	9 (100)	1.000

*Indicates significant correlation

### Interventions

The main treatments initiated for the patients consisted of antiviral therapy (113: 100%), antibiotic therapy (112: 99.1%), and corticosteroid (5: 4.4%). Also, based on oxygen support administered for the patients, the majority of non-severe patients used nasal cannula (96.8%). In addition, for severe cases, other modalities such as invasive mechanical ventilation, non-invasive ventilation, or high flow masks were used according to the condition of patients in our study. Patients using invasive mechanical ventilation had significantly higher mortality in our study (*P* < 0.01) (Table 1).

### Outcome

Based on the prognosis of the disease, an overall 8% mortality rate was documented among the patients in our study, in which the majority were among the ICU admitted patients (5: 55.6%). Also, 68 (60.2%) of our patients achieved total recovery, and 7 (6.2%) were discharged with follow-up and home isolation.

## Discussion

Based on the results of this study up to March 30, 2020, a total number of 113 patients were admitted to Shiraz hospitals, the capital of Fars province, Iran, with a diagnosis of COVID-19. The mean age of hospitalized patients was 53 years old, with a male to female ratio of 1.6:1. Of these patients, 29 (25.7%) are still hospitalized, 68 (60.2%) were discharged, 7 (6.2%) were discharged with outpatient treatment and 9 (8%) died. 11 (9.7%) cases were admitted to the ICU due to the severity of the disease.

Virological findings indicated that some Asian populations may potentially be more susceptible to Covid-19 than other races [31–33]. Chan et al. confirmed the person-to-person transmission of the virus [16]. Our results showed that COVID-19 infects men more than women; these findings are consistent with the findings of the previous studies [1, 34–36]. In early reports from China, the susceptibility of men contracting the disease was believed to have a relationship with their link to the seafood market as most workers there were men [34]. Nevertheless, as the disease spread to other countries throughout the world, this theory was weakened as men were also more susceptible to the disease in other countries. Several theories have been proposed in this respect; Li et al. reported that the male to female ratio can be attributed to the role of sex hormones and protection of the X chromosome, which plays an essential role in adaptive and innate immunity [36]. However, it may be assumed that due to Iranian culture, men tend to have more person to person contact as they work outside the house more than women who usually stay at home and do household chores.

Taking the patients’ age into consideration, most patients with severe conditions were aged over 50 years. They had comorbid diseases such as hypertension and diabetes, which is aligned with the data that has been previously reported [19, 34]. Fang et al. described a theory stating that since the coronavirus binds to its target through an angiotensin-converting enzyme 2 (ACE-2) expressed by epithelial cells in kidney, lung and blood vessels, the infection can facilitate an increased risk of developing severe COVID-19 in individuals who take ACE inhibitors and angiotensin II Type-I receptor blockers (ARBs) and also among diabetic patients as they tend to have an increase in ACE-2 expression [37–39].

Putting aside the typical symptoms such as fever, cough, and myalgia [6, 40, 41], our data revealed that many patients presented with atypical symptoms such as abdominal pain, diarrhea, nausea, vomiting, and vertigo. In a study carried out by Lechien et al. [42] in Europe, headache, loss of smell, rhinorrhea and nasal obstruction were among the most common symptoms of patients following typical symptoms. Moreover, Spinato et al. stated that out of 202 confirmed COVID-19 patients, 130 (64.4%) reported to have an altered sense of smell or taste [43]. However, due to the design of our study and incomplete hospital records these data were not collected in our study. These data suggest that aside from the focus on typical symptoms, atypical presentations of the disease must be kept in mind as most of our patients developed gastrointestinal symptoms observed in earlier diagnosis and facilitated prevention of the spread of the disease. Moreover, these differences in the presentation of the virus might be due to alterations in the virus genotype or the level of expression of the virus receptors, including ACE2, in which necessitates further studies and evaluation.

Based on our data, those who had lower O_2_ saturation on admission and presented with rales on physical examination were significantly associated with being severely ill and achieving poorer prognosis. Furthermore, those who were severely ill had lower heart rates and blood pressure. These findings can be explained by the theory that coronavirus affects not only the respiratory system but also the cardiovascular system. Based on published studies, COVID-19 patients have had high levels of myocardial injury biomarkers in their blood samples [4, 34]. Furthermore, Zheng et al. stated that this myocardial injury might be related to ACE2 which is widely expressed in the cardiovascular system as well as the respiratory system [44].

In terms of laboratory data, abnormalities included leukocytosis in 10.8%, lymphopenia in 12.6%, thrombocytopenia in 15.6%, PT and PTT in 77.9 and 45.8% were seen in the patients. Patients with severe conditions had higher increases in C - reactive protein and ESR levels, and those who died had higher levels of lactate dehydrogenase. Furthermore, severe cases of COVID-19 had more laboratory abnormalities than those who were admitted in general wards. The same results have been reported in previous studies, except for the fact that the number of cases with lymphopenia in our study was lower than other studies [1, 28, 34, 45, 46].

It is worth mentioning that in our study, the NLR ratio was significantly higher in those who were admitted to the ICU and those who died, but the average level of NLR in most patients was higher than 3.13. Therefore, we assume that a higher cut off in approach to COVID-19 patients based on the NLR ratio might be beneficial as Lie et al. reported the appropriate cutoff of 3.13 [47]. The overall results of laboratory data suggest that the novel coronavirus infection is associated with the activation of immune system responses with an impact on lymphocytes and the activation of the coagulation cascade. Thus, further studies in this area can be beneficial in the treatment of COVID-19.

Based on our data, 4 (4.9%) of our patients, who were under 50 years old and not severely ill, had normal chest CT scans. Hu et al. also reported that 29.2% of the younger asymptomatic patients in his study had normal radiologic findings [48]. Most abnormal radiologic findings consisted of ground-glass opacities, consolidation, and crazy paving presented mostly in both lungs and peripheral areas. These data, which is consistent with other publications, suggest that CT scan can play a crucial role in the diagnosis and evaluation of the severity of the disease [28, 34, 40].

The fatality rate of patients included in the current study was 8%, which is quite near to the national mortality rate in Iran which was reported to be 7% based on documented COVID-19 patients but was significantly higher compared to most studies from China [34, 49, 50], although some of which reported higher or equal mortality rate in hospitalized patients [36, 41]. Given that China had a history of SARS outbreak in 2003, they were able to successfully control the disease with the help of their previous experience and appropriate leadership. Inadequate awareness towards the disease in the early stages, lack of medical protection, high infectivity of the virus, and lack of treatment measures in Iran led to a rapid increase in the number of patients and mortality rate [51]. Moreover, since those who developed mild symptoms did not seek medical treatment, the actual mortality rate in the society might be even lower.

Controlling the source of infection, taking preventive measures, early diagnosis, isolation of suspicious cases, and supportive care have been taken into consideration to cease the spread of the virus. Although many randomized controlled trials have been initiated around the world, no specific treatment or vaccine has of yet been proposed for COVID-19. Antiviral and antibiotic therapies have been used to treat COVID-19; however, none of them were found to be properly beneficial [34, 46]. In the present study, all of the patients received antiviral therapy, all except one received antibiotic treatment, and 5 cases (4.4%) received corticosteroids.

As with any hospital-based study, this study has its own limitations. Firstly, we encountered some missing data as there were variations in patients’ documents in two hospitals due to the limited time and shortage of trained medical staff. Secondly, some patients were still being admitted to the hospital during the time of writing the manuscript, which could have affected the outcome results. Thirdly, due to the limited number of patients and given the fact that most patients with mild symptoms were not hospitalized and were not included in the study, further community-based studies are justified to explore and clarify the different aspects of this disease in Iran, as one of the most important focal points of the disease.

## Conclusion

In this multicenter case series of 113 hospitalized patients, an 8% mortality rate for the COVID-19 patients in south of Iran was reported. Some patients developed atypical symptoms at the time of admission which makes the diagnosis difficult. Finding the source of infection and studying the behavior of COVID-19 is crucial for understanding the pandemic. Furthermore, early diagnosis, improving detection methods, timely isolation, and proper treatment are the key factors in fighting this infection.

## Data Availability

SPSS data of the participants can be requested from the authors. Please write to the corresponding author if you are interested in such data.

## Abbreviations

ACE-2: Angiotensin-converting enzyme 2
ARBs: Angiotensin II Type-I receptor blockers
ARDS: Acute respiratory distress syndrome
AST: Aspartate aminotransferase
BUN: Blood urea nitrogen
CoV: Coronavirus
COVID-19: Novel coronavirus 2019
CRP: C-reactive protein
CT: Computed tomography
ESR: Erythrocyte sedimentation rate
HRCT: High-resolution Computed tomography
ICU: Intensive care unit
INR: International normalized ratio
MERS: Middle East Respiratory Syndrome
NLR: The neutrophil to lymphocyte ratio
NTC: No template control
PTT: Prolonged partial thromboplastin time
RT-PCR: Real-time reverse transcriptase-polymerase chain reaction
SARS: Severe Acute Respiratory Syndrome
SD: Standard Deviation
SPSS: Statistical Package for Social Sciences
WHO: World Health Organization
